# Assessment of Knowledge, Awareness, and Perceptions of Robotic-Assisted Surgery Among the Adult Population in the United Arab Emirates

**DOI:** 10.7759/cureus.103425

**Published:** 2026-02-11

**Authors:** Fatma Almadani, Malak Sondoqah, Razan Abdulsattar Awad, Yusur Al-Sudani, MHD Munzer Hussin Alali, Mohamed Feras Ebedin

**Affiliations:** 1 Department of Basic Medical Sciences, College of Medicine, University of Sharjah, Sharjah, ARE

**Keywords:** health education, knowledge, public awareness, robotic-assisted surgery, technology literacy

## Abstract

Background: Successful adoption of robotic-assisted surgery (RAS) requires acceptance not only from healthcare organizations that implement these technologies but also from the general public, who would ultimately undergo such procedures. This study aimed to assess public awareness, knowledge, and perceptions of RAS in the United Arab Emirates (UAE).

Materials and methods: This cross-sectional study was conducted among 433 adults aged 18 years and above residing in the UAE between September 2021 and March 2023. Participants were recruited via convenience sampling through social media platforms. Data were collected using a 20-item self-administered questionnaire assessing knowledge, awareness, and perceptions of RAS. Responses were anonymized to ensure confidentiality. Data were analyzed in SPSS Statistics version 22 (IBM Corp. Released 2013. IBM SPSS Statistics for Windows, Version 22.0. Armonk, NY: IBM Corp.) and summarized using descriptive statistics; associations between variables were assessed using inferential tests.

Results: The majority of respondents (81.1%, n = 351) demonstrated inadequate awareness of RAS. Awareness was significantly associated with occupation, education level, and technology literacy. Individuals working in medical fields showed significantly higher levels of awareness than those in non-medical fields (25.4% vs. 15.8%; p = 0.028). Participants with postgraduate education had higher awareness (30.4%) than those with bachelor's degrees (16.8%; p = 0.034). Technological literacy also played a major role: participants proficient with technology were 2.5 times more likely to have adequate RAS knowledge (p = 0.003). Respondents with adequate knowledge were 14.5 times more likely to consider undergoing RAS compared to those with inadequate knowledge (p < 0.001).

Conclusions: Public awareness of RAS in the UAE remains low and is influenced by educational background, occupation, and technological literacy. Targeted public education efforts are recommended to improve awareness and acceptance of RAS.

## Introduction

Robotic-assisted surgery (RAS) represents a major advancement in modern surgical practice and has been increasingly adopted worldwide across multiple surgical specialties. Since the introduction of robotic surgical platforms, most notably the da Vinci® Surgical System (Intuitive Surgical, Inc., Sunnyvale, CA, USA), RAS has transformed minimally invasive surgery by enhancing surgical precision, improving visualization through three-dimensional imaging, and increasing dexterity via articulated instruments [1]. These technological advantages have been associated with reduced blood loss, shorter hospital stays, faster postoperative recovery, and, in selected procedures, improved surgical outcomes compared to conventional techniques [2,3]. As a result, RAS has become an integral component of surgical care in many high-income countries and continues to expand globally.

Despite its growing clinical utilization, public understanding and awareness of RAS remain limited. Existing literature has predominantly focused on the perspectives of surgeons, medical trainees, and patients already undergoing surgical care, with relatively fewer studies assessing awareness and perceptions within the general population [4,5]. Previous international studies have demonstrated that misconceptions regarding the surgeon's role, system autonomy, safety, and cost are common, and that awareness levels are strongly influenced by educational background, occupational exposure, and technological literacy [6].

In the Middle East, and particularly in the United Arab Emirates (UAE), the adoption of RAS systems has expanded rapidly in both public and private healthcare institutions as part of national strategies aimed at advancing healthcare innovation [7]. However, data on public awareness, knowledge, and perceptions toward RAS within the UAE remain scarce. Understanding public perceptions is essential, as patient acceptance and informed decision-making are critical to the successful integration of emerging surgical technologies.

This cross-sectional study aims to examine public awareness, knowledge, and perceptions of RAS among adults in the UAE and to explore factors influencing these attitudes, including demographics, technological literacy, and media exposure.

This article was previously presented at the 6th American University in the Emirates (AUE) student research competition on September 11, 2023, and as a poster presentation at the 5th Internal Medicine Update (IMU) conference 2023 in October 2023.

## Materials and methods

Study design

This cross-sectional study was conducted between September 2021 and March 2023 to assess public awareness, knowledge, and attitudes toward RAS among adults residing in the UAE. The study was designed and reported in accordance with the Strengthening the Reporting of Observational Studies in Epidemiology (STROBE) guidelines for cross-sectional studies. The University of Sharjah Research Ethics Committee issued approval REC-22-02-16-05-S.

Study sample

Cochran's formula $N = \frac{4P(1 - P)}{ME^2}$ was used to calculate the sample size [8], assuming an awareness prevalence of 36.8%, a 95% confidence level, and a 5% margin of error, resulting in a minimum required sample size of 358 participants. To account for potential incomplete responses, the target sample size was increased to 376. A total of 462 participants were recruited, of whom 433 completed the questionnaire fully and were included in the final analysis.

Study population

Adults aged ≥18 years residing in the UAE and able to complete the questionnaire in Arabic or English were eligible to participate. Individuals who submitted incomplete questionnaires were excluded. Participants were recruited using convenience sampling through social media platforms, including WhatsApp (Meta Platforms, Inc., Menlo Park, CA, USA), Instagram (Meta Platforms, Inc., Menlo Park, CA, USA), and Telegram (Telegram FZ-LLC, UAE).

Research instrument and data collection

Data were collected using a 20-item self-administered online questionnaire adapted from a previously validated instrument developed by Buabbas et al. [6]. The questionnaire comprised three domains: demographics (seven items: gender, age, nationality, place of residence, educational level, employment status, and profession), awareness of RAS (six items), and attitudes toward RAS (seven items). Awareness and attitude items were assessed using a five-point Likert-scale response format (1 = strongly disagree to 5 = strongly agree), with higher scores indicating greater awareness and more favorable attitudes toward RAS.

The questionnaire was distributed electronically as an online survey. Participation was voluntary and anonymous, and informed consent was obtained electronically prior to participation. A copy of the distributed questionnaire is provided in the Appendices.

The questionnaire was administered in both Arabic and English. A forward-backward translation process was employed to ensure linguistic and conceptual equivalence. The questionnaire was translated from English into Arabic and independently back-translated into English by bilingual authors. Any discrepancies were reviewed and resolved through consensus to ensure semantic consistency between versions. Although the questionnaire was adapted from a previously validated instrument, a formal pilot study was not conducted. This limitation was acknowledged and considered when interpreting the findings.

Reliability

Internal consistency of the awareness domain was assessed using Cronbach’s alpha ($\alpha = \frac{k}{k - 1} \times \left[ 1 - \frac{\sum \text{item variances}}{\text{total score variance}} \right]$), which demonstrated acceptable reliability (α = 0.753) [9,10].

Statistical analysis

Data were analyzed using SPSS Statistics version 22 (IBM Corp. Released 2013. IBM SPSS Statistics for Windows, Version 22.0. Armonk, NY: IBM Corp.). Descriptive statistics were used to summarize participant characteristics. Associations were assessed using Pearson’s chi-square test or Fisher’s exact test, as appropriate, and odds ratios (ORs) with 95% confidence intervals (CIs) were calculated. Awareness and attitude scores were analyzed as continuous variables. For descriptive purposes, attitude scores were further categorized into low, moderate, and high levels to facilitate interpretability. Awareness and knowledge were categorized as adequate or inadequate. A two-tailed p-value of < 0.05 was considered statistically significant.

## Results

Sociodemographic data

A total of 433 participants were included in the study; sociodemographic data are represented in Table 1. Females comprised 59.6% (n = 258) of the sample. Participants’ ages ranged from 18 to 64 years, with a median age of 23 years. Most respondents resided in Sharjah (36.3%, n = 157) and Dubai (31.2%, n = 135). Nearly half of the sample were Non-Emirati Arabs (47.8%, n = 207), followed by Emiratis (46.2%, n = 200) and non-Arabs (6.0%, n = 26). Regarding education, 45.5% (n = 197) held a bachelor’s degree. Students represented 48.0% (n = 208) of participants, and 61.2% (n = 265) reported working in non-medical fields.

**Table 1:** Sociodemographic data of the respondents

Category	Number of participants (n)	Percentage of participants (%)
Sex		
Female	258	59.6%
Male	175	40.4%
Age		
18-30 years	261	60.6%
31-40 years	64	14.8%
41-50 years	65	15.1%
>50 years	41	9.5%
Residence		
Abu Dhabi	113	26.1%
Dubai	135	31.2%
Sharjah	157	36.3%
Other Emirates	28	6.5%
Nationality		
Emirati	200	46.2%
Non-Emirati	207	47.8%
Non-Arab	26	6.0%
Education		
School	113	26.1%
College/ diploma degree	77	17.8%
Bachelor's degree	197	45.5%
Postgraduate degree	46	10.6%
Profession		
Non-medical	265	61.2%
Medical related	54	12.5%
Medical	114	26.3%
Employment status		
Employed	188	43.4%
Unemployed	37	8.5%
Student	208	48.0%

Awareness of RAS

Awareness scores were generally low, with only 18.9% of participants meeting the predetermined cutoff for adequate awareness. Education level was significantly associated with awareness (p = 0.038). Participants with postgraduate degrees were over twice as likely to have adequate awareness compared with those holding a bachelor’s degree (30.4% vs. 16.8%; OR = 2.174, 95% CI: 1.047-4.516), as shown in Table 2.

**Table 2:** Association between education level and awareness of RAS A bachelor’s degree was used as the reference category for OR calculations. Percentages represent the distribution of awareness level within each education category (column percentages). RAS: robotic-assisted surgery, OR: odds ratio, CI: confidence interval

Education level	Adequate awareness (n, %)	Inadequate awareness (n, %)	p-value	OR (95% CI)
School	26 (23.0%)	87 (77.0%)	0.177	0.673 (0.379-1.198)
Some college/diploma degree	9 (11.7%)	68 (88.3%)	0.296	1.520 (0.690-3.348)
Bachelor’s degree	33 (16.8%)	164 (83.2%)	Reference	1.00 (reference)
Postgraduate degree	14 (30.4%)	32 (69.6%)	0.034	2.174 (1.047-4.516)

Occupation also influenced awareness (p = 0.028). Individuals working in non-medical professions were significantly less likely to have adequate awareness than those in medical fields (15.8% vs. 25.4%; OR = 0.552, 95% CI: 0.323-0.943). Nonetheless, there was no significant difference in awareness between individuals within non-medical and medical-related fields (OR = 1.258, 95% CI: 0.648-2.846), as shown in Table 3.

**Table 3:** Association between occupation and awareness of RAS A non-medical field was used as the reference category for OR calculations. Percentages represent the distribution of awareness level within each education category (column percentages). RAS: robotic-assisted surgery, OR: odds ratio, CI: confidence interval

Occupation field	Adequate awareness (n, %)	Inadequate awareness (n, %)	p-value	OR (95% CI)
Medical	29 (25.4%)	85 (74.6%)	0.028	0.552 (0.323-0.943)
Non-medical	42 (15.8%)	223 (84.2%)	Reference	1.00 (reference)
Medical-related	11 (20.4%)	43 (79.6%)	0.416	1.258 (0.648-2.846)

In the multivariable logistic regression analysis, participants identifying as proficient in technology were 2.5 times more likely to have adequate awareness compared with those reporting only basic technological literacy (32.1% vs. 15.8%; adjusted OR = 2.526, 95% CI: 1.365-4.674). In contrast, there was no significant difference in the proportion of individuals with adequate awareness between those with intermediate literacy and those with basic literacy (15.2% vs. 15.8%; OR = 0.952, 95% CI: 0.527-1.721). Similarly, individuals with below-basic literacy did not differ significantly from those with basic literacy (23.1% vs. 15.8%; OR = 0.624, 95% CI: 0.161-2.421), as shown in Table 4.

**Table 4:** Association between technological literacy and awareness of RAS A basic literacy level was used as the reference category for OR calculations. Percentages represent the distribution of awareness level within each education category (column percentages). RAS: robotic-assisted surgery, OR: odds ratio, CI: confidence interval

Technological literacy	Adequate awareness (n, %)	Inadequate awareness (n, %)	p-value	OR (95% CI)
Below basic	3 (23.1%)	10 (76.9%)	0.493	0.624 (0.161-2.421)
Basic	27 (15.8%)	144 (84.2%)	Reference	1.00 (reference)
Intermediate	25 (15.2%)	140 (84.8%)	0.872	0.952 (0.527-1.721)
Proficient	27 (32.1%)	57 (67.9%)	0.003	2.526 (1.365-4.674)

Knowledge of RAS principles

**Figure 1:**
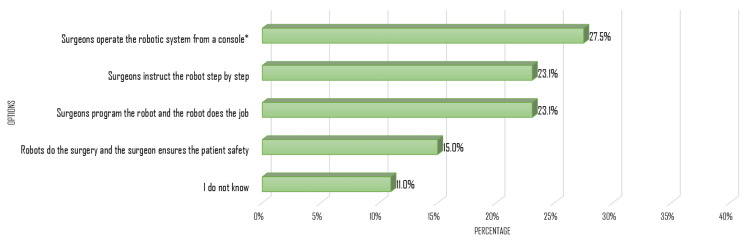
Participants’ responses to the definition of RAS Correct response is marked by an asterisk (*). RAS: robotic-assisted surgery

When asked to identify the correct definition of RAS, only 27.5% selected that RAS involves a surgeon operating the robotic system from a console. The majority (61.2%) selected incorrect responses, indicating substantial misunderstanding of RAS fundamentals, as shown in Figure 1.

**Figure 2:**
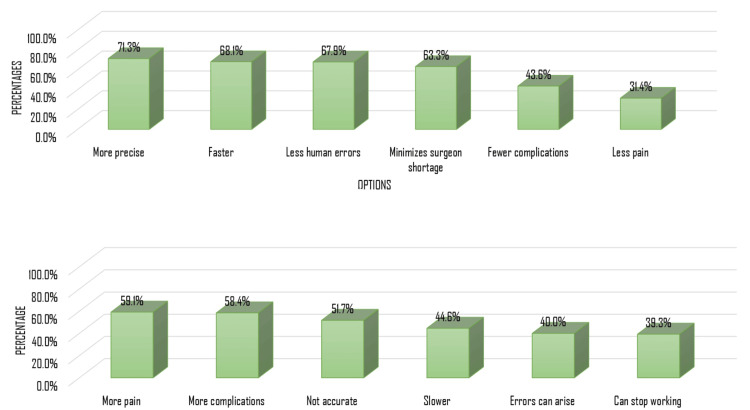
Participants’ perceptions of the (A) advantages and (B) disadvantages of RAS RAS: robotic-assisted surgery

Regarding participants’ perceptions of the advantages of RAS (Figure 2A), awareness was moderate. Most participants recognized improved surgical precision (71.3%), shorter operative times (68.1%), reduced human error (67.9%), and reduced surgeon shortages (63.3%) as benefits. Fewer participants identified reductions in complications (43.6%) or postoperative pain (31.4%) as advantages, indicating partial understanding of clinical benefits.

Knowledge of potential disadvantages (Figure 2B) was similarly limited. While many participants acknowledged increased pain (59.1%), higher complication rates (58.4%), and potential inaccuracy (51.7%) as risks, a substantial proportion were less aware of slower procedures (44.6%), possible errors (40.0%), or technical malfunctions (39.3%), reflecting gaps in awareness of RAS limitations.

Attitudes toward RAS

Although awareness was low, attitudes toward RAS were generally moderate. Based on the predefined attitude categories, 47.6% of participants demonstrated a high attitude, reflected by a willingness to undergo RAS; 34.2% showed a moderate attitude (unsure); and 18.3% exhibited a low attitude (unwilling).

**Figure 3:**
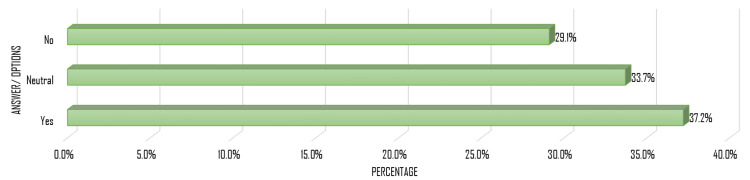
Participants’ willingness to recommend RAS RAS: robotic-assisted surgery

Participants with adequate awareness had significantly higher odds of a high attitude toward RAS than those with inadequate awareness (adjusted OR = 14.5, 95% CI: 5.35-29.29), as shown in Table 5. Regarding the recommendation of RAS, 37.2% indicated they would recommend it to others, 29.1% would not, and 33.7% remained unsure (Figure 3).

**Table 5:** Association between awareness of RAS and attitudes The neutral likelihood of undergoing RAS was used as the reference category for OR calculations. Percentages represent the distribution of awareness level within each education category (column percentages). RAS: robotic-assisted surgery

Likelihood to undergo RAS	Adequate awareness (n)	Inadequate awareness (n)	p-value	OR (95% CI)
Extremely unlikely	3	49	0.613	1.408 (0.371-5.338)
Unlikely	5	75	0.65	1.293 (0.425-3.932)
Neutral/unsure	10	116	Reference	1.00 (reference)
Likely	49	99	<0.001	5.741 (2.764-11.927)
Extremely likely	15	12	<0.001	14.500 (5.352-29.287)

## Discussion

This study reveals that public awareness of RAS in the UAE remains low, with fewer than one in five participants demonstrating adequate knowledge. Findings indicate that education level, occupation, and technological literacy are key determinants of awareness and acceptance, highlighting the need for targeted public education initiatives and improved access to reliable information tailored to the UAE context [11,12].

Higher levels of education, particularly postgraduate study, were associated with greater awareness of RAS. This aligns with existing literature indicating that individuals with higher levels of education are more likely to be exposed to scientific innovations and possess greater health and technology literacy [13,14]. In contrast, participants with only a bachelor’s degree demonstrated lower levels of knowledge, suggesting gaps in public dissemination of information about RAS. In the UAE, where higher education is expanding but not universally health- or technology-focused, integrating medical and technological literacy across diverse programs may be critical for fostering public understanding.

Consistent with previous research, healthcare workers exhibited significantly higher awareness of RAS than those in non-medical careers. Their direct exposure to surgical technologies likely contributes to their greater familiarity. Limited awareness among non-medical participants suggests unequal access to information and emphasizes the need for broader community education efforts [15]. Public health campaigns tailored to non-medical audiences may help bridge this gap and promote equitable understanding of emerging medical technologies.

Technological proficiency appeared to influence awareness. Participants with higher digital literacy were significantly more knowledgeable about RAS. This reflects global trends, indicating that individuals who are comfortable with technology are more open to innovations in healthcare and better able to understand complex technological systems [16]. Given the UAE’s rapidly digitizing healthcare sector, initiatives to improve general digital literacy could enhance both awareness and acceptance of RAS. Notably, social media was the predominant source of RAS information, underscoring the importance of optimizing digital platforms to ensure accurate, accessible, and culturally relevant health communication.

Despite generally positive attitudes toward RAS, participants expressed notable concerns regarding the reliability and safety of robotic systems. Many reported fears of potential malfunctions, findings consistent with studies conducted in other regions. These concerns may stem from limited public understanding of RAS safeguards and intraoperative control mechanisms. Cost was also a frequently cited concern. Participants perceived RAS as more expensive than traditional surgical approaches, echoing prior research suggesting that cost remains a major barrier to the broader adoption of RAS [15,16]. Addressing these concerns through targeted public education, particularly by emphasizing system safety, clinical outcomes, and long-term benefits, such as reduced complications and faster recovery, can strengthen public trust and acceptance.

A strong relationship was observed between participants’ knowledge levels and their willingness to undergo RAS. In the present study, individuals with adequate knowledge were markedly more likely to consider RAS than those with inadequate knowledge. This reinforces the existing literature showing that informed patients are more likely to choose advanced medical technologies when they understand their safety, effectiveness, and potential benefits [17,18]. As public understanding of RAS's precision, minimal invasiveness, and shorter recovery periods improves, acceptance and demand for RAS are likely to increase.

Recommendations

The findings of this study highlight the critical importance of expanding public education to improve awareness and acceptance of RAS. Although RAS offers many advantages, persistent misconceptions related to safety, reliability, and cost continue to limit public confidence. To address these gaps, healthcare institutions and policymakers should prioritize digital outreach, including social media campaigns that provide accurate, accessible information. Additionally, community education programs are advisable to address common misconceptions, particularly when combined with collaboration with educational institutions to enhance technological and health literacy. These efforts will play a vital role in enhancing public understanding and supporting the effective integration of robotic technology into healthcare systems [19,20].

Strengths and limitations

This study’s strengths include a large, geographically diverse sample across multiple Emirates, providing a broad overview of public awareness of RAS in the UAE. Additionally, it features a structured questionnaire with acceptable reliability and enables standardized data collection and comparison with the existing literature.

Limitations include convenience sampling via social media, which may limit generalizability, introduce recruitment bias, and favor tech-literate participants, thereby inflating awareness scores. The absence of a formal pilot study limits the ability to confirm the questionnaire’s reproducibility. Additionally, the extended data collection period (2021-2023) may have introduced temporal variation, as public awareness could have shifted due to new hospital marketing campaigns or government health initiatives. Furthermore, the association between attitudinal status toward RAS and participants’ demographic backgrounds was not analyzed in this study. Examining these relationships in future research would provide valuable insights for understanding factors influencing attitudes toward RAS. Despite these limitations, the findings provide valuable insight into public knowledge and perceptions of RAS in the UAE, offering a foundation for targeted education and outreach initiatives.

## Conclusions

This cross-sectional study demonstrates that public awareness of RAS among adults in the UAE remains limited, despite generally positive attitudes toward its use. Awareness was significantly influenced by educational attainment, professional background, and technological literacy, highlighting persistent disparities in access to and understanding of advanced surgical technologies. Importantly, individuals with adequate knowledge were substantially more willing to undergo RAS, underscoring the critical role of public education in shaping acceptance and informed decision-making.

As robotic technologies continue to expand within the UAE healthcare system, targeted public education initiatives are essential to address misconceptions regarding safety, reliability, and cost. Improving technological and health literacy may enhance patient trust, autonomy, and readiness to adopt innovative surgical options. Future studies employing probability-based sampling and longitudinal designs are recommended to assess causal relationships better and evaluate the impact of educational interventions on public awareness and acceptance of RAS.

## Appendices

**Table 6:** Questionnaire on awareness and attitudes toward RAS RAS: robotic-assisted surgery, UAE: United Arab Emirates

Section name	Item number	Question	Choices
Demographics	A1	What is your gender?	Female
			Male
	A2	What is your age?	--- years old
	A3	What is your nationality?	Emirati
			Non-Emirati Arab
			Non-Arab
	A4	What is your current place of residency?	Abu Dhabi/Al Ain
			Dubai
			Sharjah
			Ajman
			Ras Al Khaimah
			Fujairah
			Umm Al Quwain
			Outside the UAE
	A5	What is your highest educational level?	Did not enter school
			Elementary school (any grade 1-6)
			Middle school (any grade 7-9)
			High school (any grade 10-12)
			Technical school
			Some college or diploma degree
			Bachelor's degree (University)
			Post-graduate degree
			Others (please specify)
	A6	What is your current employment status?	Employed full-time
			Employed part-time
			Self-employed
			Unemployed
			Student
			Retired
			Others (please specify)
	A7	What is your field of work?	Medical
			Non-medical
			Medical-related
Awareness of RAS	B1	How comfortable are you in using technology devices?	Very uncomfortable
			Uncomfortable
			Neutral
			Comfortable
			Very comfortable
	B2	What is your level of literacy in technology?	Non-literate
			Below basic
			Basic
			Intermediate
			Proficient
	B3	Have you heard of RAS before?	Yes
			No
	B4	If you answered the previous question with Yes, please specify your source:	Internet/social media
			Friends
			Family members/relatives
			Family doctor
			Not sure
			Others (please specify)
	B5	How aware are you of RAS?	Not at all aware
			Slightly aware
			Somewhat aware
			Moderately aware
			Extremely aware
	B6	As per your knowledge, how do you think RAS works?	I don’t know
			Surgeons use robotic arms
			Surgeons instruct the robots step by step
			Surgeons program the robot,nd the robot does the job
			Robots do the surgery and the surgeon ensure the patient’s safety
Attitude toward RAS and its advantages and disadvantages	C1	Regarding the advantages of RAS, please rate your level of agreement with the following statements?	Options are represented in Table 7.
	C2	Regarding the disadvantages of RAS, please rate your level of agreement with the following statements?	Options are represented in Table 8.
	C3	If you needed to do surgery, what is the likelihood that you would agree to do RAS?	Extremely unlikely
			Unlikely
			Neutral/unsure
			Likely
			Extremely likely
	C4	What is the likelihood that you would consider recommending RAS as a surgical option to a relative or friend?	Extremely unlikely
			Unlikely
			Neutral/unsure
			Likely
			Extremely likely
	C5	Are you with or against the use of RAS? Explain why?	-----
	C6	Do you think there is a need to implement an educational program on RAS?	Yes
			No
	C7	Are you interested in knowing more about RAS?	Not interested at all
			Not interested
			Indifferent
			Interested
			Very interested

**Table 7:** Advantages of RAS RAS: robotic-assisted surgery

Statement	Strongly disagree	Disagree	Somewhat disagree	Neutral (not sure)	Somewhat agree	Agree	Strongly agree
Advantages of RAS							
Less pain							
Fewer complications							
Is faster							
It is more precise							
Minimizes surgeons' shortage							
Fewer human errors							

**Table 8:** Disadvantages of RAS RAS: robotic-assisted surgery

Statement	Strongly disagree	Disagree	Somewhat disagree	Neutral (not sure)	Somewhat agree	Agree	Strongly agree
Disadvantages of RAS							
More pain							
More complications							
Is slower							
It can stop working							
Errors can arise							
Not accurate							
